# The neuroanatomy of social trust predicts depression vulnerability

**DOI:** 10.1038/s41598-022-20443-w

**Published:** 2022-10-06

**Authors:** Alan S. R. Fermin, Toko Kiyonari, Yoshie Matsumoto, Haruto Takagishi, Yang Li, Ryota Kanai, Masamichi Sakagami, Rei Akaishi, Naho Ichikawa, Masahiro Takamura, Satoshi Yokoyama, Maro G. Machizawa, Hui-Ling Chan, Ayumu Matani, Shigeto Yamawaki, Go Okada, Yasumasa Okamoto, Toshio Yamagishi

**Affiliations:** 1 Center for Brain, Mind and Kansei Sciences Research, Hiroshima University, 1-2-3 Kasumi, Minami-ku, Hiroshima, Hiroshima 734-8553 Japan; 2 School of Social Informatics, Aoyama Gakuin University, Kanagawa, Japan; 3 Brain Science Institute, Tamagawa University, Tokyo, Japan; 4 Department of Neuroinformatics, Araya Inc., Tokyo, Japan; 5 Department of Psychiatry and Neurosciences, Hiroshima University, Hiroshima, Japan; 6 Social Value Decision Making Unit, Riken Center for Brain Science, Saitama, Japan

**Keywords:** Social behaviour, Social neuroscience, Predictive markers, Psychiatric disorders

## Abstract

Trust attitude is a social personality trait linked with the estimation of others’ trustworthiness. Trusting others, however, can have substantial negative effects on mental health, such as the development of depression. Despite significant progress in understanding the neurobiology of trust, whether the neuroanatomy of trust is linked with depression vulnerability remains unknown. To investigate a link between the neuroanatomy of trust and depression vulnerability, we assessed trust and depressive symptoms and employed neuroimaging to acquire brain structure data of healthy participants. A high depressive symptom score was used as an indicator of depression vulnerability. The neuroanatomical results observed with the healthy sample were validated in a sample of clinically diagnosed depressive patients. We found significantly higher depressive symptoms among low trusters than among high trusters. Neuroanatomically, low trusters and depressive patients showed similar volume reduction in brain regions implicated in social cognition, including the dorsolateral prefrontal cortex (DLPFC), dorsomedial PFC, posterior cingulate, precuneus, and angular gyrus. Furthermore, the reduced volume of the DLPFC and precuneus mediated the relationship between trust and depressive symptoms. These findings contribute to understanding social- and neural-markers of depression vulnerability and may inform the development of social interventions to prevent pathological depression.

## Introduction

Major depressive disorder (MDD) is a pervasive mental health condition that affects millions of people worldwide^1–3^. Social issues substantially contribute to the development of MDD, including diverse matters such as income inequality, gender, and racial discrimination, violence, harassment, parental separation, child abuse, social conflict, and social isolation^4–13^. Given the burden that aversive social interactions cause on mental health, several studies have attempted to identify whether and which social personality traits operate as premorbid risk factors for depression vulnerability. Individual differences in social personality traits, such as high neuroticism, agreeableness, and extraversion or low concern about others’ welfare and low trust in others have been shown to predict future depressive states and symptoms, including pathological depression^14–31^. At the biological level, despite the well-established functional and anatomical neurobiology of MDD^32–38^, only a few studies have investigated the neural substrates underlying the link between social personality traits and the development of depression^31,39,40^, but no study has addressed whether the neurobiology of trust plays a role in the expression of depressive symptoms.

Trust, a social personality trait linked with the cognitive ability to analyze social cues and estimate others’ trustworthiness, such as whether to expect reciprocal cooperation or observance of social norms, plays a fundamental role in the quality of interpersonal relations^41,42^. Estimating others’ trustworthiness and actual trust behavior are important not only for the initiation and maintenance of daily social relations but also impact large-scale issues such as political representation, military coalitions, international economic trade, and stability of democracies^41–45^. Trust influences not only the fabric of social relations, but the lack and breach of trust exert substantial negative effects on public welfare and mental health^28,42,46,47^. For instance, following an influential suggestion that lack of trust disrupts well-being^28^, multiple studies have consistently linked low levels of trust with MDD across different cultures^29,30,48–52^. At the biological level, several studies have investigated the genetic^53–55^, hormonal^56–58^, neuroanatomical^59–61^, and neurofunctional bases of trust^62–64^. There have also been attempts to identify abnormalities in the neural control of trust in patients clinically diagnosed with psychiatric disorders^62,65,66^, although it remains unknown whether similar abnormalities in the neurobiology of trust may also be present in healthy individuals with a non-clinical diagnosis of depression but exhibiting depressive symptoms.

Our previous studies have identified several social and psychological factors associated with trust^42,67–76^. Our studies on the neurobiology of trust have also shown an association between trust and volume of the amygdala with a polymorphism of the oxytocin hormone^54,55^. Despite the significant impact that trust exerts on mental health and social relations, often culminating in a significant personal and social cost, including health problems such as depression, work burnout, and break of social relationships^29,46,47,77,78^, it remains unclear whether the neurobiology of trust underlies its association with mental health. More specifically, it remains unknown whether low trust is associated with abnormally reduced gray matter volume of brain structures previously observed in MDD patients and linked with the degree of currently experienced depressive symptoms. To elucidate this issue and understand how the neuroanatomy of trust may be linked with the development of psychiatric disorders, the present study sought to investigate whether the neuroanatomy (e.g. regional gray matter volume) of trust was linked to individual differences in the severity of depressive symptoms in healthy human subjects with no formal clinical diagnosis of MDD. Determining not only the link between trust and mental health but also whether its underlying neurobiological substrates contribute to depression vulnerability may help advance early social and non-invasive neural interventions to combat and prevent the development of pathological depression.

One important psychological factor our research discovered is that high trusters are more accurate at recognizing and using social cues to evaluate the risk of engaging in interpersonal relations with potential aversive outcomes, whereas low trusters tend to overestimate the risk and negative outcomes of being deceived by others and avoid social interactions with uncertain outcomes^42,69,72,73^. These findings suggest that low trusters may exhibit increased anxiety due to the possibility of being taken advantage of by others when leaving oneself in a vulnerable situation that the decision to trust others presents. Furthermore, the constant use of a social avoidance strategy to protect oneself against others’ selfish behaviors suggests that low trusters may participate in a smaller social network, while high trusters may have an expanded social network. Previous studies have demonstrated a significant link between depression, high anxiety, and reduced social participation^79–83^. In light of our previous results and the latter findings, we also investigated the association of trust with social anxiety and social network size.

To identify the neuroanatomical basis of the relationship between trust and depression vulnerability, we used magnetic resonance imaging to acquire gray matter (GM) volume data from a large sample of healthy participants from a large-scale study (Tamagawa Sample) conducted at Tamagawa University (Tokyo, Japan). We used psychological questionnaires to assess individual differences in trust, social anxiety and social network size and a psychiatric questionnaire where participants self-reported about their current degree of experienced depressive symptoms. High self-reported depressive symptoms were used as an indicator of depression vulnerability. Furthermore, to reliably demonstrate that brain regions linked to trust and depressive symptoms are related to actual neuroanatomical abnormalities commonly observed in MDD, we also investigated GM volume abnormalities in patients clinically diagnosed with MDD from another large-scale study (Hiroshima Sample), conducted at Hiroshima University Hospital (Hiroshima, Japan).

## Results

### Results 1: low trust linked with depression vulnerability, social anxiety and social network size

To investigate the relationship between trust and depressive symptoms, trust was measured twice with the 5-item Yamagishi general trust attitude scale^42^ (see “Methods”). The first assessment of trust (TA1) was performed about 17 months before the assessment of depressive symptoms and the second (TA2) was performed about 6 months prior to it. Depressive symptoms were measured with the Beck Depression Inventory I (BDI-I) and BDI-II scales which are psychiatric scales commonly used to establish a prognosis of pathological depression^84,85^. The precedence of trust and neuroanatomical data acquisition was important to examine the predictive power of these measures for subsequent depressive symptoms of the same subjects. Unless otherwise specified, here we present all statistical analyses using the BDI-II scores as this scale was commonly used in the Tamagawa and Hiroshima samples and has been revised to address new criteria for clinical diagnosis of MDD.

**Figure 1:**
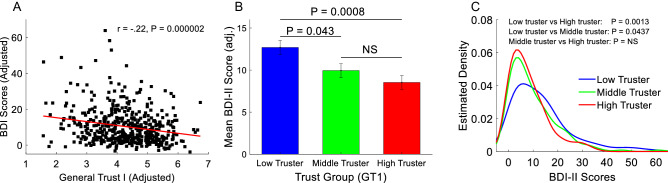
Trust and depressive symptoms. **(A)** Significant negative correlation between trust (TA1) and depressive symptoms measured up to 17 months apart. **(B)** Significant higher depressive symptoms in low trusters relative to middle and high trusters. The three groups were created based on their trust scores (TA1). **(C)** The distribution of depressive symptoms among low trusters was significantly skewed toward higher values relative to middle and high trusters.

Pearson correlation analyses (two-tailed, controlling for age, sex, education and income) revealed significant negative correlations between TA1 and BDI-II scores (*r* = –0.22, *P* = 0.000002, Fig. 1A) and between TA2 and BDI-II scores (*r* = –0.14, *P* = 0.0025). Social anxiety also showed significant negative correlation with TA1 (*r* = –0.16, *P* = 0.0006) and TA2 (*r* = –0.13, *P* = 0.0074). Positive correlations were found between social network size and TA1 (*r* = 0.11, *P* = 0.0197) and TA2 (*r* = 0.17, *P* = 0.0006). The two trust measurements (TA1 and TA2) were also significantly positively correlated (*r* = 0.64, *P* < 0.0000001), an association that also remained significant after controlling for age, sex, education, and income (*r* = 0.6, *P* < 0.0000001).

To demonstrate the robustness of the relationship between trust and depressive symptoms, participants were classified into three groups based on their TA1 scores (low trusters: bottom 33.33%, middle trusters: middle 33.33%, and high trusters: top 33.33%). An ANCOVA (controlling for age, sex, education and income) found a significant group effect on BDI-II scores (*F*[2,463] = 7.07, *P* = 0.0009, Table S1). A post-hoc analysis (Bonferroni corrected with a statistical threshold of *P*_*Bonf*_ < 0.05) revealed significantly higher BDI-II scores among low trusters relative to middle trusters (*P*_*Bonf*_ = 0.0482, Fig. 1B) and high trusters (*P*_*Bonf*_ = 0.0007, Fig. 1B), but not between middle trusters and high trusters (*P*_*Bonf*_ = 0.5020). Similar findings were observed when trust group classification was based on TA2 (Table S1). Significant differences in the distribution of BDI-II scores were observed between low trust and high trust groups (*k* = 0.2243, *P* = 0.0007, Fig. 1C), a marginally significant difference between low trust and middle trust groups (*k* = 0.1424, *P* = 0.0783), but not between middle trust and high trust groups (*k* = 0.09, *P* = 0.5084). Altogether, these findings demonstrate that low trust is significantly associated with high depressive symptoms, high social anxiety and smaller social network size.

### Results 2: the neuroanatomy of trust

We next sought to investigate the neuroanatomical substrates of trust with a focus on the GM volume of brain regions previously implicated in social cognition and trust, namely the middle frontal gyrus (as a representative of the dorsolateral prefrontal cortex, DLPFC), dorsomedial prefrontal cortex (DMPFC), ventromedial prefrontal cortex (VMPFC), temporal parietal junction (TPJ: supramarginal and angular gyri), precuneus, posterior cingulate cortex (PCC), and amygdala^31,62,64,86–97^.

Regional GM volumes of social brain regions were extracted using an automated neuroimaging parcellation method based on neuroanatomical landmarks provided by the Neuromorphometrics brain atlas (see “Methods”, Table S2). Pearson correlations (two-tailed, controlling for age, sex, and total intracranial volume—TIV) revealed an association between increased GM volume of social brain regions with higher trust scores. Significant positive correlations (following False Discovery Rate, FDR, correction) were found between trust (TA1) and GM volumes of the left DLPFC (r = 0.11, *P*_*FDR*_ = 0.0394), right DLPFC (r = 0.12, *P*_*FDR*_ = 0.0394), left DMPFC (r = 0.18, *P*_*FDR*_ = 0.0020), right DMPFC (r = 0.13, *P*_*FDR*_ = 0.0210), left PCC (r = 0.12, *P*_*FDR*_ = 0.0340), left precuneus (r = 0.17, *P*_*FDR*_ = 0.0020), and right precuneus (r = 0.16, *P*_*FDR*_ = 0.0033). Similar findings were observed between the second assessment of trust (TA2) and GM volume of social brain regions (Table 1).

**Table 1:** Social brain regions linked with trust and depressive symptoms.

Area name	Hemisphere	Atlas name	General trust			General trust			Depressive symptoms		
			GT1			GT2			BDI-II		
			*r-coeff *	*P-unc.*	*P-FDR *	*r-coeff *	*P-unc.*	*P-FDR *	*r-coeff *	*P-unc.*	*P-FDR *
**Amygdala nuclei **											
Amygdala	Left	lAmy	0.08	0.0879	0.1256	0.03	0.4790	0.5322	−0.08	0.0821	0.1263
Amygdala	Right	rAmy	0.06	0.1744	0.2180	−0.01	0.8288	0.8724	−0.07	0.1802	0.2253
**Dorsolateral prefrontal cortex**											
**Middle frontal gyrus**	Left	lMidFroGy	0.11	**0.0138**	**0.0394**	0.13	**0.0060**	**0.0200**	-0.15	**0.0021**	**0.0210**
**Middle frontal gyrus**	Right	rMidFroGy	0.12	**0.0124**	**0.0394**	0.18	**0.0002**	**0.0040**	-0.14	**0.0042**	**0.0210**
**Dorsomedial prefrontal cortex**											
**Superior medial frontal gyrus**	Left	lSupMedFroGy	0.18	**0.0001**	**0.0020**	0.14	**0.0036**	**0.0144**	-0.11	**0.0250**	**0.0455**
Superior medial frontal gyrus	Right	rSupMedFroGy	0.13	**0.0042**	**0.0210**	0.11	0.0179	0.0511	-0.07	0.1757	0.22525
**Ventromedial prefrontal cortex**											
Frontal pole	Left	lFroPo	0.09	0.0545	0.0838	0.07	0.1285	0.2336	-0.02	0.6980	0.7756
Frontal pole	Right	rFroPo	0.10	0.0283	0.0515	0.07	0.1720	0.2646	-0.11	**0.0203**	**0.0426**
Rectus gyrus	Left	lRecGy	0.06	0.2080	0.2334	0.05	0.3073	0.4097	-0.09	0.0539	0.0898
Rectus gyrus	Right	rRecGy	0.11	**0.0171**	**0.0428**	0.10	0.0404	0.0898	-0.05	0.3034	0.3569
Ventromedial frontal area	Left	lMedFroCbr	0.02	0.7430	0.7430	0.01	0.8810	0.8810	-0.01	0.7844	0.8109
Ventromedial frontal area	Right	rMedFroCbr	0.07	0.1557	0.2076	0.05	0.3342	0.4178	-0.01	0.8109	0.8109
**Temporo-parietal junction**											
Angular gyrus	Left	lAngGy	0.10	0.0390	0.0650	0.09	0.0640	0.1280	-0.11	**0.0213**	**0.0426**
Angular gyrus	Right	rAngGy	0.10	0.0279	0.0515	0.14	**0.0029**	**0.0144**	-0.11	**0.0187**	**0.0426**
Supramarginal gyrus	Left	lSupMarGy	0.05	0.3083	0.3245	0.07	0.1584	0.2640	-0.12	**0.0122**	**0.0407**
Supramarginal gyrus	Right	rSupMarGy	0.06	0.2101	0.2334	0.04	0.4261	0.5013	-0.07	0.1593	0.2253
**Posterior cingulate-precuneus**											
**Posterior cingulate cortex**	Left	lPosCinGy	0.12	**0.0085**	**0.0340**	0.10	0.0402	0.0898	-0.12	**0.0105**	**0.0407**
Posterior cingulate cortex	Right	rPosCinGy	0.10	0.0264	0.0515	0.06	0.2001	0.2859	-0.14	**0.0036**	**0.0210**
**Precuneus**	Left	lPCu	0.17	**0.0002**	**0.0020**	0.17	**0.0005**	**0.0050**	-0.14	**0.0030**	**0.0210**
**Precuneus**	Right	rPCu	0.16	**0.0005**	**0.0033**	0.16	**0.0012**	**0.0080**	-0.11	**0.0202**	**0.0426**

Partial correlations showing the relationship between gray matter volume of social brain regions, trust and depressive symptoms. All analyses controlled for effects of age, sex, education, income and TIV. Brain regions with names highlighted in bold font are significantly associated with both trust and depressive symptoms. P-values in bold survived FDR correction.

**Figure 2:**
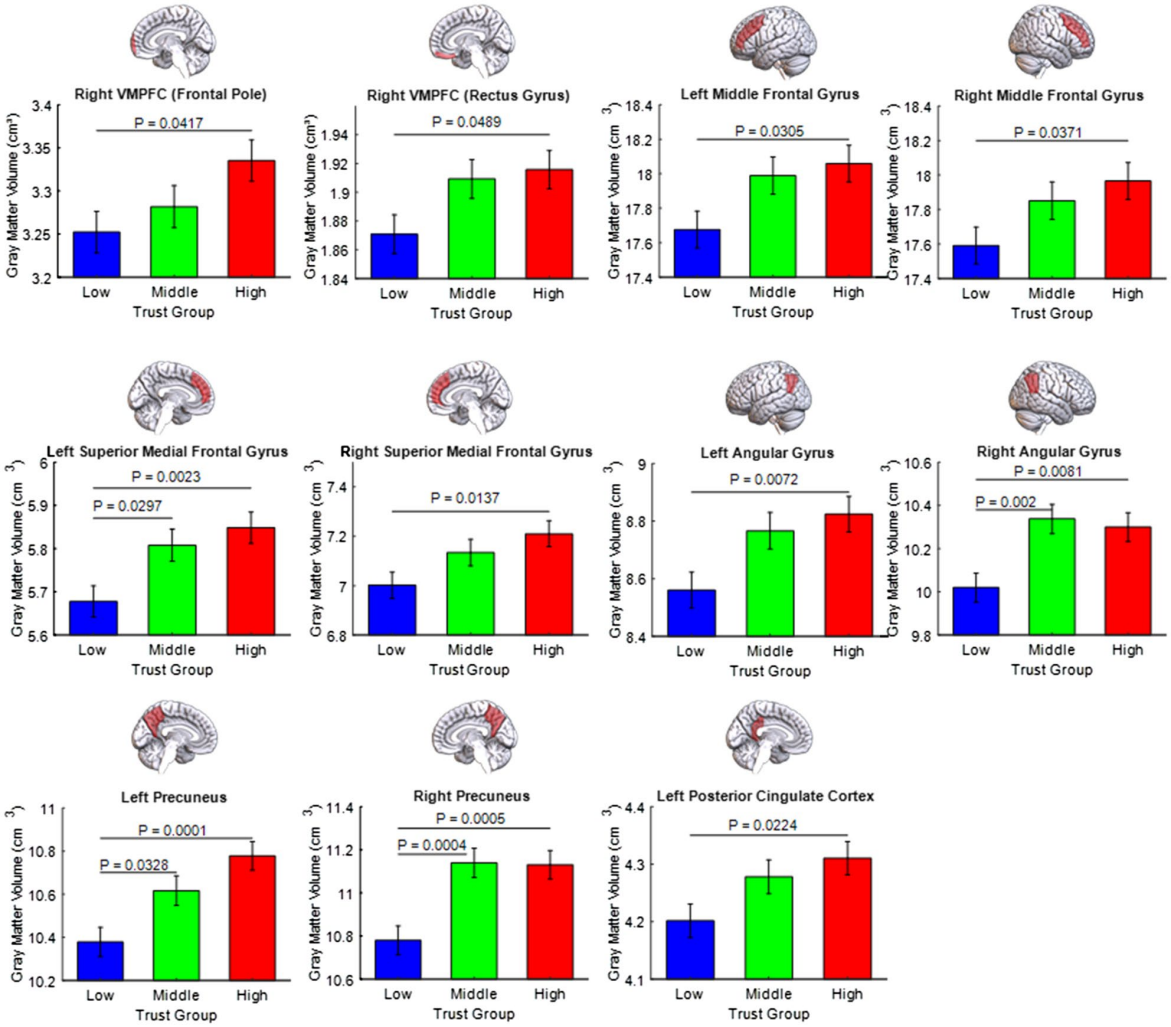
Reduced volume of social brain regions among low trusters. Social brain regions positively associated with trust (Table 1) also showed robust group differences when participants were classified as low trusters, middle trusters and high trusters. Population marginal means and error bars (standard error) were calculated with ANCOVA and all P-values are Bonferroni corrected for multiple comparisons. Gray matter volume is adjusted for age, sex, education, income and TIV.

Analysis of covariance with trust group as a factor (low trusters, middle trusters, high trusters), also demonstrated a significant relationship between trust level and GM volumes of social brain regions. Low trusters showed significant GM volume reduction, relative to high trusters, in the left angular gyrus (*P*_*Bonf*_ = 0.0072), right angular gyrus (*P*_*Bonf*_ = 0.0081), left DLPFC (*P*_*Bonf*_ = 0.0305), right frontal pole (*P*_*Bonf*_ = 0.0417), right rectus gyrus (*P*_*Bonf*_ = 0.0489), left DLPFC (*P*_*Bonf*_ = 0.0371), right DLPFC (*P*_*Bonf*_ = 0.0371), left DMPFC (*P*_*Bonf*_ = 0.0023), right DMPFC (*P*_*Bonf*_ = 0.0137), left PCC (*P*_*Bonf*_ = 0.0224), left Precuneus (*P*_*Bonf*_ = 0.0001) and right Precuneus (*P*_*Bonf*_ = 0.0005) (Fig. 2, Table S3).

**Figure 3:**
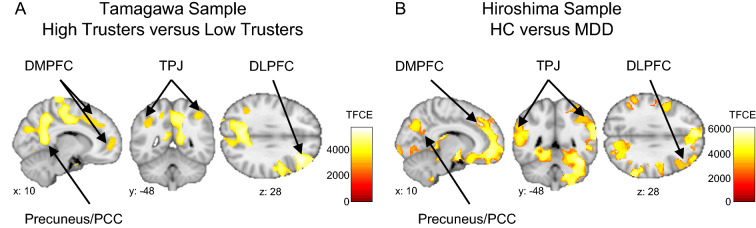
Whole-brain VBM analyses of trust and depressive symptoms. **(A)** Social brain regions with increased gray matter volume in high trusters relative to low trusters. Highlighted brain regions include the MFG, DMPFC, precuneus, posterior cingulate and angular gyrus. **(B)** Enlargement of social brain regions in healthy controls relative to MDD patients in the Hiroshima sample. Both analyses were performed using a non-parametric permutation method and a statistical threshold of P_FWE_ < 0.05, family-wise error corrected for the whole-brain.

A whole-brain voxel-based morphometry (VBM) analysis (controlling for age, sex, education, income and TIV) using a permutation method also demonstrated significant enlargement of social brain regions among high trusters relative to low trusters, including the DLPFC, DMPFC, PCC, precuneus and amygdala (*P*_*FWE*_ < 0.05, corrected for the whole-brain) (Fig. 3A, Table S4).

### Results 3: the neuroanatomy of trust linked with depressive vulnerability

High depressive symptoms (BDI-II scores) were significantly associated with reduced GM volumes of social brain regions associated with trust (Table 1). Trust brain regions with reduced GM volumes linked with high depressive symptoms included the left DLPFC (*r* = –0.15, *P*_*FDR*_ = 0.0210), right DLPFC (*r* = –0.14, *P*_*FDR*_ = 0.0210), left DMPFC (*r* = –0.11, *P*_*FDR*_ = 0.0455), right VMPFC (frontal pole, *r* = –0.11, *P*_*FDR*_ = 0.0426), left angular gyrus (*r* = –0.11, *P*_*FDR*_ = 0.0426), right angular gyrus (*r* = –0.11, *P*_*FDR*_ = 0.0426), left supramarginal (*r* = –0.12, *P*_*FDR*_ = 0.0407), left PCC (*r* = –0.12, *P*_*FDR*_ = 0.0407), right PCC (*r* = –0.14, *P*_*FDR*_ = 0.0210), left precuneus (*r* = –0.14, *P*_*FDR*_ = 0.0210) and right precuneus (*r* = –0.11, *P*_*FDR*_ = 0.0426). These findings indicate that reduced GM volumes of social brain regions, especially of the bilateral DLPFC, left DMPFC, left PCC and bilateral precuneus, are linked with both low trust and high depressive symptoms.

### Results 4: social brain structures linked with MDD in the Hiroshima sample

Despite an interval of about 17 months between acquisition of brain structure data to administration of the BDI-II scale in the Tamagawa Sample, the above neuroanatomical results are consistent with previous findings in which both depressive symptoms and brain structure data were acquired on the same day^98–100^. However, in order to reliably demonstrate that reduced GM volumes of social brain regions associated with both low trust and high depressive symptoms in the Tamagawa Sample may represent a feature of a depressed brain, we conducted a whole-brain VBM analysis to investigate GM volume differences between healthy controls (HC) and MDD patients in the Hiroshima Sample. This analysis revealed significant GM volume reduction among MDD patients, relative to HC, in social cognitive brain areas including the DLPFC, DMPFC, VMPFC, PCC, precuneus, TPJ, insula, amygdala (P_FWE_ < 0.05, corrected for the whole-brain) (Fig. 3B, Table S5).

### Results 5: DLPFC and precuneus volumes mediate the relationship between trust and depression vulnerability

**Figure 4:**
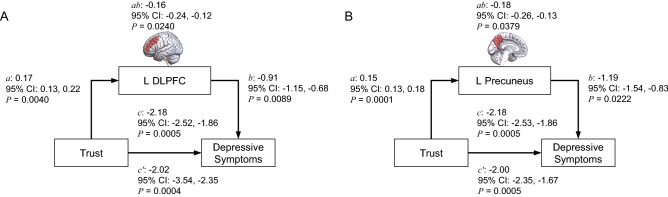
Mediation role of social brain regions on the link between trust and future depressive symptoms. Mediation analysis revealed significant indirect effects of GM volume of the left DLPFC **(A)** and left precuneus **(B)** on the relationship between trust and future depressive symptoms.

Given that GM volumes of social brain regions showed a consistent relationship with both trust and depressive symptoms (Table 1), we performed mediation analyses to investigate whether volumes of social brain regions served a mediation function in the relationship between trust and depressive symptoms. In these analyses we treated trust (TA1) as the independent variable, BDI-II scores as the dependent variable and GM volumes of social brain structures as mediators of the relationship between trust and depressive symptoms (see “Methods”). These analyses revealed significant mediation effects of the volume of the left DLPFC (indirect path *ab coeff*: –0.16; *confidence interval*: –24, –12; *P* = 0.0240) and volume of the left precuneus (indirect path *ab coeff*: –0.18; *confidence interval*: –26, –13; *P* = 0.0379) on the relationship between trust and depressive symptoms (Fig. 4, Table S6).

## Discussion

The present study investigated the underlying neuroanatomy of trust and its association with depression vulnerability measured as the degree of self-reported depressive symptoms in a sample of healthy participants. We found a previously unknown association between GM volume of brain regions linked with both trust and depressive symptoms. Low trust was significantly associated with reduced GM volumes of brain regions implicated in social cognition, including the dorsolateral and dorsomedial PFC, TPJ, PCC and precuneus. Strikingly, reduced GM volumes of the same brain regions associated with low trust were also associated with high depressive symptoms in our healthy sample and were also observed in a sample of MDD patients when compared to health controls. Furthermore, our analyses also demonstrated that GM volumes of the left DLPFC and precuneus mediated the relationship between trust and depressive symptoms.

The present findings, demonstrating a significant association between low trust and high depressive symptoms in our Japanese sample, suggest that low trusters exhibit greater vulnerability to depression and are consonant with those of previous studies linking low trust with MDD in different cultures, such as the United States^29^, Korea^48^, China^49^, South Africa^50^, Sweden^51^ and Finland^52^. Our results also add to the vast literature demonstrating a link between multiple social personalities and vulnerability to development of depression^5,31,101–104^ and suggest that low trust toward others may be used as a reliable biosocial marker to predict depression vulnerability across different cultures.

The main finding of the present study was the demonstration of a previously unknown association that the GM volumes of social brain regions linked with low trust are also associated with high depressive symptoms. Our structural neuroimaging analyses revealed that both low trust and high depressive symptoms are linked with reduced GM volumes of the bilateral angular gyrus, bilateral DLPFC, bilateral DMPFC, bilateral precuneus, VMPFC (right frontal pole and right rectus gyrus) and left PCC. The whole-brain VBM analysis also revealed a negative relationship between trust and GM volume of the parahippocampus-amygdala region. The causes of volume reductions of social brain regions linked with both low trust and high depressive symptoms have yet to be identified. Since the present study did not longitudinally track the aversive social experiences contributing to reduced trust and a consequent increase in depressive symptoms, we cannot establish a causality between low trust and an increase in depression vulnerability. Also, since none of our participants in the Tamagawa sample have been formally diagnosed with MDD, we cannot confidently attribute the reduced GM volumes of social brain regions linked with low trust and high depressive symptoms to possible neurodegenerative processes reported in MDD^105–111^. Furthermore, to date only one longitudinal epidemiological study has attempted to establish a causal link between low trust and future diagnosis of MDD^29^. Thus, we will limit our following discussion on individual differences in brain structure linked with both low trust and high depression vulnerability to genetic or experience-driven neural plasticity processes.

Genetic processes may explain neuroanatomical differences linked to low trust and high depressive symptoms as demonstrated by studies showing heritability of GM volume in humans, including prefrontal and posterior cingulate cortices, and amygdala^112–114^. Family studies also suggest a genetic factor in heritability of brain structure and development of depression. For instance, neuroimaging studies have shown that family relatives at high risk of depression also exhibit abnormalities in the volumes of social brain structures similar to those observed in family members with MDD^115–119^. In support of this genetic interpretation, our work and others have shown that individual differences in trust and in amygdala volume, a region identified in the present study, have been associated with oxytocin receptor gene (OXTR) polymorphism^53–55^. However, future studies are needed to investigate whether and what genetic polymorphisms may underlie the link of reduced GM volumes of social brain regions with low trust and depression vulnerability.

Experience-dependent neuroanatomical plasticity driven by the use of distinct social strategies may also explain the reduced GM volumes of social brain regions associated with both low trust and high depressive symptoms. For instance, behavior studies link higher trust with types of experiences in childhood, levels of intelligence in early adolescence, and with higher accuracy in adulthood at recognizing social cues and evaluating the risk of engaging in social relations, quick learning of other’s past behaviors in order to respond appropriately, exploration of new relationships, and learning richer models of a partner’s behaviors^42,120–129^. In line with this social experience-dependent view of neuroanatomical plasticity, social brain regions with reduced GM volumes linked with low trust and high depressive symptoms in the current study have been implicated in several aspects of social cognition and experience, such as social deliberation in DLPFC^87,89,93,95^, moral reasoning and valuation in the VMPFC^130–132^, theory-of-mind and empathy in the DMPFC and TPJ^133,134^, switching and focusing attention to social context in the PCC^135–137^, self-perspective and in-group attitude in the precuneus^138^, as well as in realization of trust-based learning and decision-making^57,59–61,63,64,139–141^. Neuroanatomical plasticity and enlargement of social brain regions, such as the prefrontal and cingulate cortices and the amygdala, has been reported in monkeys ascending social group hierarchy^91^ and in humans undergoing social mental training^142^. Based on these findings, it is reasonable to speculate that higher use of the functions of social brain regions by high trusters may trigger volume enlargement of these regions and may support higher resilience to development of depressive symptoms. Further studies may help determine how social experiences, specifically those that rely on trust-based cognitive processes, lead to either enlargement or reduction in GM volumes of social brain regions and contribute to depression vulnerability.

How dysfunctions in trust-based cognitive processes contribute to vulnerability and development of depressive symptoms remains obscure. Here, we focus on the negative social bias that characterizes low trust, which is the constant expectation of aversive outcomes from uncertain social interactions^42^. We speculate that this constant negative bias to hypothetical future aversive events resembles rumination or repetitive thoughts about experienced distressful events that predict development of depression and that are frequently observed in MDD patients^143–145^. This negative bias may lead low trusters to exhibit higher social anxiety and avoidance of social interactions, which may contribute to development of depressive symptoms, such as reduced mood and motivation, sense of discouragement, unhappiness and loss of social interest (as measured by the BDI scale). In support of this view, studies with adolescents have shown an association of low trust and high rumination with depressive symptoms^146,147^. Our present findings also revealed a significant association of low trust with high social anxiety and reduced social network. Given the association between anxiety, social network and depression^148,149^, one could argue that the development of higher levels of depressive symptoms, such as observed among low trusters, may be facilitated by their high social anxiety, reduced social networks and consequent reduced access to social support.

Trust has been associated with other personality traits with distinct cognitive characteristics that have been linked with depression, such as neuroticism, extraversion and egocentrism^150–153^. Among these personalities, neuroticism is characterized by high suspicion about other’s intentions, which is similar to low trusters’ constant expectation of other’s untrustworthy behaviors. Thus, while trust-based cognitive processes may contribute to depression vulnerability, we cannot rule out cognitive processes shared between trust and other personality traits which may be present within the same individual. Future studies are needed to demonstrate how trust and other cognitive processes, such as rumination or neuroticism, contribute to social isolation and development of depressive symptoms.

Understanding how trust-related psychological processes, e.g., negative bias and estimation of trustworthiness, and brain structures interact to contribute to depression vulnerability is a work in progress. A candidate explanation comes from our mediation analyses showing that, among the 11 brain regions associated with both trust and depression vulnerability, the link between low trust and high depressive symptoms was significantly mediated by the reduced volume of only two social brain regions, the DLPFC and precuneus (Fig. 4). As described above, functions of the DLPFC and precuneus have been implicated in trust-based processes, model-based decision-making, social deliberation, and theory-of-mind^93,95,133,138,154–157^. The social functions of the DLPFC and precuneus can be interpreted under the more integrative approach of the active inference framework, which suggests that the brain uses generative models to make predictions of expected sensory data^158–160^. According to active inference models, failures in the generation of context-based predictions (e.g. results of own actions or the behavior of others) and in the estimation of confidence of those predictions or in updating of generative models (e.g. stored representations of other’s behavior patterns) at the different levels of the neural hierarchy may contribute to the vulnerability and development of depression^161–163^. Based on the active inference framework, we speculate that reduced volumes of the DLPFC and precuneus among low trusters may weaken their social predictive functions and impair the learning or updating of social models. Thus, the increased depression vulnerability of trusters may be associated with the generation of suboptimal social predictions, such as an exaggerated negative bias in the form of a constant expectation of aversive outcomes to yet to happen social interactions, and lower social exploration given their increased social anxiety and reduced social network. Consistent with this active inference interpretation of trust, poor cognitive processes, such as those observed among low trusters, have been implicated in the development and neuropathology of depression^86,164–179^. In addition, transcranial magnetic stimulation, neurofeedback, and functional neuroimaging studies suggest that strengthened activity and connectivity of the DLPFC and precuneus decrease the severity of depressive symptoms observed in MDD patients^37,180–183^. Overall, our findings suggest that continuous, poor use of social cognitive processes by low trusters, possibly due to weakening of social predictive functions, especially of the DLPFC and precuneus due to their reduced gray matter volume, may facilitate depression vulnerability.

In conclusion, the present study revealed that reduced GM volumes of social brain regions mediate the relationship between low trust and high depressive symptoms. Despite restricting our analyses to neuroanatomical brain data in healthy participants, the neuroanatomical abnormalities observed among low trusters resembled those of MDD patients in the present study and also the functional and connectivity dysfunctions in social brain regions reported in previous studies with MDD patients^37,38,182–184^. The present findings may inform social policies, behavioral and non-invasive neural interventional strategies that may be used to increase social trust, restore social predictive and cognitive processes, and reduce depression vulnerability. For instance, higher behavioral trust is observed following administration of oxytocin by nasal spray^57^, and the balance of oxytocin in the brain has been suggested as a potential treatment for anxiety and depression^185^. The use of cognitive behavioral therapy, previously shown to modulate the activity of social brain regions and improve self-reported quality-of-life in subthreshold depression patients^184^ may also be used as a social cognitive intervention to increase social trust and prevent depression. Finally, the successful demonstration that learning to control the neural activity of social brain regions by neurofeedback training can reduce the severity of depressive symptoms^182,183,186^ also suggests the potential use of neurofeedback methods to prevent depression vulnerability in low trusters. Our findings demonstrate that neuro-social markers comprised of social personality trait and neuroanatomical information may enable early identification of individuals at higher risk of depression and development of preventive therapeutical interventions.

## Methods and materials

### Tamagawa sample, data acquisition and analysis

Both behavioral and MRI studies were conducted at the Brain Science Institute of Tamagawa University. The study protocol was approved by the Tamagawa University Brain Science Institute Ethics Committees, and all experiments were conducted in accordance with the approved protocol, which met requirements of the Declaration of Helsinki. Written informed consent was provided by each participant prior to participation in the study.

The data and methods used to select participants and process structural neuroimaging data have been reported in details in our previous studies^95,187^. Six hundred non-student residents living in and around Machida, a suburb of Tokyo, were selected from a list of approximately 1670 applicants who responded to a brochure that had been distributed to roughly 180,000 households. Following invitation, only 564 (F = 290, M = 273) participated in the initial wave of the study in which demographic data and structural neuroimaging data were collected. One participant was excluded from the study for inconsistent responses to demographic items. Of the remaining 563 participants, we acquired valid brain imaging data of 470 participants. Participants visited the lab in several waves to answer questionnaires and participate in behavior experiments. See Table S7 for the timeline of data reported in the present study.

Trust was measured with the 5-item, 7-point Yamagishi scale^42^, which includes the items: (i) most people are basically honest; (ii) generally, I trust others; (iii) most people are basically good-natured and kind; (iv) most people trust others; (v) most people are trustworthy. Social anxiety was measured with the Social Interaction Anxiety Scale^188^. Social network size was measured with the Cohen’s social ties questionnaire^189^.

High-resolution T1-weighted neuroanatomical images were acquired using a 3 T Siemens Trio A Tim MRI scanner and rapid acquisition gradient echo (MPRAGE) sequence (TR = 2000 ms; TE = 1.98 ms; field of view = 256 × 256 mm; number of slices = 192; voxel size = 1 × 1 × 1 mm; average = 3 times).

Brain structural T1-weighted images were processed and analyzed using the Computational Anatomy Toolbox (CAT12, http://dbm.neuro.uni-jena.de/cat/) and Statistical Parametric Mapping software (SPM12, http://www.fil.ion.ucl.ac.uk/spm). Images were bias-corrected, tissue-classified (gray matter, GM; white matter, WM; and cerebral spinal fluid, CSF), and registered using linear (12-parameter affine) and non-linear transformations (warping) within the CAT12 default pre-processing pipeline. This initial step generated modulated normalized data that were then smoothed via the standard SPM12 smoothing pipeline with a full-width at half-maximum smoothing kernel of 8 × 8 × 8 mm. Overall GMV, WMV, CSF volume, and total intracranial volume (TIV) were then obtained using the CAT12 TIV estimation function. CAT12 uses the Neuromorphometrics brain atlas for volume estimation of cortical and subcortical brain areas in native space. Details of this neuroanatomical parcellation process can be found in the CAT12 software.

Estimated Neuromorphometrics ROI GM volumes were used to perform statistical. We used Matlab to perform partial correlations to investigate the relationship of ROI GM volumes with trust and depressive symptoms. Given the large dataset used in the present study, all behavior analyses controlled for the effect of age, sex, education and income, whereas relationships with ROI GM volumes included an additional control for TIV.

Analysis of Covariance (ANCOVA) with Matlab was used to investigate group differences (low trust, middle trust, high trust) in regional GM volumes. These analyses included as covariates age, sex, education, income and TIV. Group differences in depressive symptoms (altruistic × selfish; low trust, middle trust and high trust) were also investigated with ANCOVA and included as covariates age, sex, education and income.

A whole-brain VBM analysis was conducted to group differences (low trust × high trust) in GM volumes. This analysis controlled for effects of age, sex, education, income and TIV. Voxel clusters reached significance if they survived statistical cluster correction (*P*_*FWE*_ < 0.05). Given the significant results found with parcellation data, we further used small-volume correction at the peak voxel of a ROI (as described in the “Introduction”) with a statistical threshold of *P*_*FWE*_ < 0.05. The statistical SPM model generated in the above analysis was then used in a whole-brain VBM permutation test using the threshold-free cluster enhancement (TFCE) method implemented by the TFCE toolbox (http://www.neuro.uni-jena.de/tfce/). Results were considered significant if clusters exceeded a whole-brain correction threshold of *P*_*FWE*_ < 0.05.

Mediation analyses were performed to identify whether social brain regions linked with both trust and depressive symptoms (Table 1) contributed to the link between trust and future depressive symptoms. In these analyses we used the Mediation Toolbox (https://canlab.github.io/repositories/) and implemented a bootstrap method with 10,000 iterations treating each volume of a social brain region as a mediator, the level of trust as the independent variable, and the degree of depressive symptoms as the dependent variable.

### Hiroshima sample, acquisition and data analysis

Patients with MDD (n = 81) and healthy controls (HC, n = 104), all right-handed Japanese citizens, were included in the study (Supplementary Table ST1). Patients participating in this study were outpatients at Hiroshima University Hospital or other medical institutions located in Hiroshima, Japan. Newspaper advertisements were used to recruit HC participants with no previous history of psychiatric disorders. Diagnosis of MDD was performed by an expert clinician following criteria according to the Diagnostic and Statistical Manual of Mental Disorders, Fifth Edition (DSM-5). In order to increase diagnosis validity, the Mini-International Neuropsychiatric Interview (MINI) was also administered to patients and HC participants to confirm the absence of other psychiatric disorders. The Beck Depression Inventory-II (BDI-II) was administered to all participants. The study followed the 1975 Helsinki Declaration of ethics principles for research involving humans and was approved by the Ethics Committee of Hiroshima University. Participants were required to sign a written informed consent form and received financial compensation for their participation. Structural MR images of Hiroshima Data were obtained using a 3 T Siemens Verio scanner with following parameters (MPRAGE, TR = 2300 ms; TE = 2.98 ms; field of view = 256 × 256 mm; number of slices = 176; voxel size = 1 × 1 × 1 mm).

Structural images were segmented into gray matter, white matter, cerebrospinal fluid, and normalized (1 × 1 × 1 voxel size) into a template space using standard parameters implemented in the Computational Neuroanatomy Toolbox (CAT12). Modulated normalized images were then smoothed with an 8 × 8 × 8 mm FWHM kernel using Statistical Parametric Mapping (SPM) software. Following CAT12 standard procedures, regional gray matter volume parcellation was performed in native space before normalization with the Neuromorphometrics Brain Atlas.

A whole-brain VBM analysis was conducted to investigate group differences (HC x MDD) in GM volume. This analysis controlled for effects of age, sex, and TIV. Voxel clusters reached significance if they survived statistical cluster correction (*P*_*FWE*_ < 0.05). Given the significant results found with the parcellation data that revealed trust group differences in ROI GM volume, we further used small-volume correction at the peak voxel of an identified ROI (as described in the introduction) with a statistical threshold of *P*_*FWE*_ < 0.05. The statistical SPM model generated in the above analysis was then used in a whole-brain VBM permutation test using the threshold-free cluster enhancement (TFCE) method implemented by the TFCE toolbox (http://www.neuro.uni-jena.de/tfce/). Results were considered significant if cluster survived a whole-brain correction threshold of *P*_*FWE*_ < 0.05.

## Supplementary Information

Supplementary Tables.

## Data Availability

The datasets used and/or analysed during the current study available from the corresponding author on request.
